# PERMA Well-Being Model and Profiler: Arabic Translation and Validation Among the Saudi Adult Population

**DOI:** 10.3390/healthcare13243185

**Published:** 2025-12-05

**Authors:** Ebtesam Abdullah Alzeiby, Aljawharh Ibrahim Alsukah, Nourah Abdulrhman AlGadheeb, Ali Faris Alamri, Monira Abdulrahman Almeqren, Uzma Zaidi

**Affiliations:** 1 Department of Psychology, College of Education and Human Development, Princess Nourah bint Abdulrahman University, Riyadh 11671, Saudi Arabia; eaalzoaby@pnu.edu.sa (E.A.A.); aialsogeh@pnu.edu.sa (A.I.A.); naalgdeb@pnu.edu.sa (N.A.A.); maalmgrn@pnu.edu.sa (M.A.A.); 2 Family Medicine, King Abdullah bin Abdulaziz University Hospital, Princess Nourah bint Abdulrahman University, Riyadh 11671, Saudi Arabia; dr.amri@hotmail.com; 3 Graduate Nursing, Wagner Hall 313, College of Nursing, South Dakota State University, Brookings, SD 57007, USA

**Keywords:** PERMA profiler, PERMA model, psychometrics, well-being, Saudi adults

## Abstract

**Background/Objective:** Psychological scales hold significant importance, measuring cultural sensitivity, experiences, values, and emotional expressions while controlling individuals’ biases regarding certain cultures. Arabic is used in a widespread, predominantly Middle Eastern and North African region, but it has diverse expressive variations. Therefore, the value of translating and validating scales lies in their compatibility with cultural norms. This study focuses on measuring the psychometric properties and factorial structure of the widely used PERMA-Profiler to assess the general well-being of the Saudi adult population. **Methods**: Using stratified sampling, (N = 2927) Saudi adults were recruited via various electronic and social media. **Results:** The confirmatory factor analysis showed strong evidence of the instrument’s validity, confirming well-being as a single latent factor composed of five dimensions with an acceptable degree of reliability. The correlation coefficients indicate considerable internal consistency for numerous factors, including positive emotions, engagement, relationships, meaning, accomplishment, health, and happiness (α = 0.83, 0.79, 0.77, 0.84, 0.80, 0.69, 0.71), respectively. The Split-Half coefficient demonstrated significant reliability (0.87). **Conclusions:** The validity and reliability of the scale are supported, confirming that PERMA can be applied to measure the well-being of Saudi society. Furthermore, the Arabic version of PERMA can be utilized in counseling and psychotherapy practice as well as in research studies. It could be a helpful tool for exploring and preventing mental health issues and improving well-being within the community.

## 1. Introduction

Translation and validation studies are of prime importance, especially when the concepts are connected to emotional expression, cultural sensitivity, and word expressions. A sound standardized measure supports the provision of normative comparison and helps to measure certain concepts for diverse populations as well. All these issues become more significant in psychology, counseling, and clinical psychology-related research. One example of such efforts is reported in a study written in the Arabic language, which focused on a limited sample of neighboring regions [1]. In addition to the generalization issue, the expression of the Arabic language and its understanding differ by region. Thus, we aimed to assess the psychometric features of the PERMA-Profiler based on Seligman’s well-being theory in Saudi Arabia by administering the Arabic translated version for Saudi adults. The study included adults from all 13 Saudi Arabian regions to provide fully representative data, various cultural contexts, and develop norms that could be used for the prevention of mental health at the community level. The project will later be enhanced for various populations, including senior citizens, to provide standardization and norms for the PERMA profiler.

Prosperity or psychological well-being are modern terms that have emerged with the evolution of positive psychology and the development of the concepts of psychological well-being, positive behavior, and good mental health [2]. These ideas hold promise in the theory of well-being, emphasizing that mental health is a multidimensional state of high levels of pleasure. Psychological well-being refers to positive mental health resulting from the existence of meaning in life, the formation of positive relationships, feelings of competence, self-esteem, positive integration, and contribution to the happiness of others [3].

Hence, there is a positive correlation between psychological well-being and healthy behavior, physical health, the educational environment, and the performance of personnel [4]. Improving quality of life is a primary concern in numerous intellectual, social, and political spheres, and interest in this topic has resulted in the creation of instruments for assessing populations’ welfare [5]. Most of the existing models of well-being address the dimension of self-well-being, which is based on the hedonic and eudaimonic factors; these are crucial for maintaining the balance of well-being [6].

The concept of well-being or prosperity has been explored by both researchers and Positive Psychology specialists. Therefore, different models of well-being have emerged based on individual researchers’ theoretical orientation with regard to well-being. Some have defended instantaneous happiness or pleasure through experiences of specific emotional states and the satisfaction of desires [7,8], while some have focused on the existence of meaning and purpose [9]. Early well-being theories included the theory of authentic happiness, which assumed that happiness is achieved through one of three pathways: the path of pleasure, a focus on positivity, emotions, the path of sharing and creating a state of flow, and the path of meaning involving a sense of purpose.

Building upon these theories, Seligman created the PERMA (positive emotions, engagement, relationships, meaning, and accomplishment) framework, a holistic approach to defining well-being. Seligman [10] identified five components that people seek due to their intrinsic stimulation and contribution to well-being. These elements should be realized, defined, and measured separately [10].

PERMA profiler is the central theoretical model in positive psychology, and its five components create a system that can be used to predict the prosperity of nations, communities, organizations, and groups [11]. The literature has revealed a good predictive association between PERMA components and health status, job satisfaction, and a good detector of psychological stress [4,11].

According to Seligman, the PERMA model is not a theory but a framework, providing guidelines for the methods or building blocks to consider when attempting to improve one’s well-being. Its five measurable factors can be used to actively develop subjective well-being in a therapeutic setting [12]. As an evidence-based model of the active components of well-being, it provides a documentary-based framework based on scientific evidence to improve psychological well-being in various areas [13,14].

There was a need for a tool that combines various constructs of well-being. Therefore, an instrument was developed by Julie Butler and Margaret Kern [15] from the University of Pennsylvania. It measures the five factors described above, as well as negative emotions and health. Butler and Kern [15] confirmed the standardization of the profiler after conducting extensive studies. The instrument was included in its initial form with one hundred and ninety-nine questions and was tested using several samples; following a series of psychometric tests, fifteen main questions were selected in the PERMA model, and then eight more questions were added. Thus, in its final form, the instrument consists of twenty-three questions. Its reliability was assessed by examining its content and visual validity [15].

Cross-cultural studies were also conducted, and different cultures were compared in the adaptation methods. The scale’s reliability was verified in studies such as that of Iasiello et al. [16], with regard to samples originating from the United States, South Australia, New Zealand, and the United Kingdom. The results indicated that South Australia had higher levels of well-being than all of the other cultures. Meanwhile, Zheng et al. [17] compared an American sample with a Chinese one. Petersen et al. [18] also aimed to apply the PERMA instrument to different samples in Germany, Norway, and New Zealand. Khaw et al. [19], from the University of Pennsylvania, conducted a cross-cultural study comparing an American sample with a Malaysian sample; the level of the Malaysian sample was lower than that of the American sample for all dimensions.

PERMA was also adapted to other variables and age groups in different countries and cultures where levels of validity and reliability were high, such as Colombia [20], Ecuador [21], Hong Kong [22], Indonesia [23], and Italy [14]. Bartholomaeus et al. [24] also aimed to adapt and apply PERMA to different age samples in Australia, with a sample of 1963 participants aged between 18 and 65 years. The results showed reasonable levels of validity and reliability and highly positive associations between the total scale and the five factors of the PERMA instrument.

Chaves et al. [25] aimed to adapt the PERMA instrument into Spanish by applying it to a sample of 23,723 university students with an average age of 21.50, in addition to 2783 employees with an average age of 36.08. The results showed high levels of validity and reliability, and the correlation was high among PERM’s results and variables of well-being in general, positive impact, and overall life happiness; physical well-being was found to be marginally linked with the growth mentality. Additionally, the confirmatory factor analysis demonstrated that the sample of employees and students had a high degree of congruence with the five factors of the PERMA model [25].

Adapting the PERMA profiler into different environments and languages is of interest to many researchers and other interested parties around the world. Table 1 summarizes some of the existing PERMA adaptation studies.

The literature shows that the PERMA profiler has been translated and validated across numerous countries and languages. Therefore, the current study aims to provide a standardized Arabic version of PERMA that can be utilized in counseling and psychotherapy, as well as research. It may also benefit the community to use it as a local norm at a preventive level. The general objective of this study is to evaluate the psychometric properties of the PERMA-Profiler in a sample of the Saudi Adult population. Thus, we formulated the following hypothesis: “there will be a positive correlation between the five factors of the PERMA-Profiler, Arabic language translated scale (positive emotions, engagement, relationships, meaning, and accomplishment) and overall well-being among a sample of Saudi adult participants.” The findings of this study will reveal whether the Arabic translation of the PERMA profiler can measure psychometric properties and the factorial structure of well-being among Saudi adults.

## 2. Materials and Methods

### 2.1. Participants

**Figure 1:**
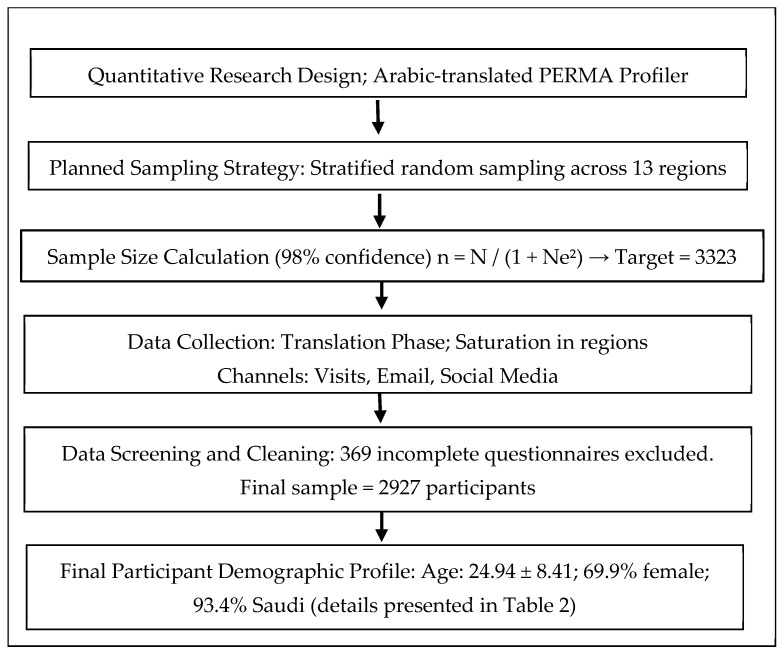
Methodological flowchart.

Our study utilized a quantitative methodology and employed an Arabic-translated PERMA profiler as the research instrument. The data collection was based on the scale distributed to the respondents to accumulate sufficient information pertaining to the objectives of the study. The intention was that the sample would be collected via stratified random sampling, in which it was divided into thirteen regions based on the administrative division of the regions of the Kingdom of Saudi Arabia. The sample size fully representative of Saudi society was calculated by the following equation: n = N/(1 + Ne^2^) [35]. N is the number of members of the study population, which is equal to (32,175,224) according to the General Authority for Statistics in the Kingdom [36]. e represents the confidence score or significance levels. By applying this equation, the sample size obtained (N = 3323) was kept above 2500, ensuring that a 98% confidence level was achieved [37]. However, during the data collection stage, saturation of data was applied to reach the target sample among the thirteen regions. All the surveys were collected individually by trained data collectors (via a visit to the home or workplace), by email, or through social media. Therefore, a certain number of (369) incomplete questionnaires were excluded (See Appendix A). The final study sample consisted of 2927 individuals with an average age of 24.94 and a standard deviation of 8.41. The percentage was 69.9% for females and 30.1% for males. Figure 1 contains a flowchart showcasing the methodology.

The characteristics of the sample according to demographic variables are described in Table 2.

### 2.2. Measures

The PERMA profiler consists of 23 questions. Queries related to health, negative feelings, loneliness, and general happiness showed no correlation with the five domains of the PERMA profiler; therefore, for CFA, 15 PERMA items were used—3 per factor—with the scale developer recommending that all of the full-scale questions be used. The instrument was arranged in the form of seven groups including various questions from the five factors of the PERMA profiler, and they must be presented in the same order; they are also answered through an 11-point response format with starting and ending points for each group anchoring the scale from 0 to 10 (never–always or not at all–completely). The average of the results for each component’s three items produces a factor score between zero and ten (with higher scores indicating greater well-being). An overall well-being score is computed via data analysis by averaging the scores of all items. Subsequently, subscale scores are determined by averaging the three items comprising each subscale.

#### Cross-Cultural Translation of PERMA Profiler

The initial configuration of the instrument was obtained through the official website of Pennsylvania University (www.authentichappiness.org), which allowed the instrument to be used for non-commercial research or evaluation purposes. Then, the instrument was translated from English into Arabic through the following steps:

1. The scale was translated from English to Arabic by two faculty members who were bilingual experts, native Arabic speakers, and had psychology doctorates.
2. The translation was reviewed by two faculty members, who were translation experts and held doctorates in psychology, and were not involved in Step 1 of the PERMA profiler translation. The review confirmed the accuracy and clarity of the translation, with some modifications made to some item statements.
3. The reverse translation from Arabic to English was conducted by a translation specialist to ensure the clarity of the translation.
4. The reverse translation was revised by two faculty members who were psychology specialists, fluent in the English language, and different from the two members responsible for the translation and the review. The reverse translation was similar to the original scale in English, which confirmed the accuracy of the translation.
5. The practical application of the scale ensured the clarity of translation, and it was carried out in two stages:

The scale was administered to ten university students by using the metacognitive interview “Think Aloud”, where the participant was asked to “think-aloud” as they answered the questions. This approach mitigates potential challenges associated with comprehending the intended significance. The final version of the validation procedure was generated based on the results of the metacognitive interview.

### 2.3. Procedure and Ethical Considerations

The study was submitted to the Institutional Review Board (IRB) of Princess Nourah bint Abdulrahman University for ethical approval (the IRB registration number with KACST was KSA: HAP-O1-R-O59). The study was granted exemption status (log number: 22-0275). However, participants received a written consent form which included a statement about voluntary participation, confidentiality, and their privacy of their information. The survey included a sociodemographic section and the Arabic version of the PERMA instrument. The order of the questionnaires was the same for all participants, starting with the consent form, then the sociodemographic questions, followed by the PERMA instrument. The data was processed through an open-source survey application (RedCap). Data collectors were trained to apply the instrument, and they helped to read out the questions for those participants who were not able to read by themselves.

### 2.4. Statistical Analysis

Every analysis was performed using SPSS (version 28), with the exception of confirmatory factor analyses, which were carried out using LISREL 8.8. The results were descriptively evaluated in relation to population norms. In order to examine the reliability indicators of the scale, Cronbach’s alpha and the Spearman–Brown Split-Half were computed using the Statistical Package for the Social Sciences (SPSS 28). For CFA model testing, LISREL8.8 software was implemented. Chi-Square (*χ*^2^), the Goodness-of-fit index (GFI), the Tucker–Lewis Index (TLI), the Normed Fit Index (NFI), the Incremental Fit Index (IFI), the Comparative Fit Index (CFI), the Relative Fit Index (RFI), the Standardized Root Mean Square Residual (SRMR), and the root mean square error of approximation (RMSEA) were evaluated [38].

## 3. Results

The Results section is organized by three interrelated psychometric themes, including internal consistency, confirmatory factor analysis (CFA), and reliability, to reflect the sequential logic and methodological rigor required in scale validation. Each theme addresses a different dimension of measurement quality, and together they provide a comprehensive evaluation of the Arabic PERMA profiler.

### 3.1. Internal Consistency

Internal consistency assesses the degree to which items within each PERMA domain perform cohesively. This step is foundational because it verifies whether items intended to measure the same construct (e.g., positive emotions) are statistically correlated; item-to-total and item-to-factor correlations reveal whether each item contributes meaningfully to its domain. Furthermore, Pearson’s Correlation offers a quantitative summary of internal coherence. This analysis ensured that each subscale of the PERMA profiler was conceptually and statistically unified before proceeding to more advanced structural modeling. Internal consistency was assessed using Pearson’s Correlation as a reliability metric for both the subscales and overall well-being, as shown in Table 3.

Table 3 indicates that the values of the correlation coefficients for the internal consistency of the PERMA Profile ranged between 0.853 and 0.873 for the positive emotions factor, 0.653 and 0.766 for engagement, 0.745 and 0.816 for relationships, 0.857 and 0.874 for meaning, and 0.662 and 0.821 for accomplishment. It ranged between 0.101 and 0.786 for negative emotions and between 0.778 and 0.873 for health. These are all significant values. Additionally, the values of the correlation coefficients of internal consistency between each item of the PERMA Profile and the overall well-being score are statistically significant. As can be seen from the previous table, the values of the correlation coefficients between the factors of the PERMA Profile (positive feelings, engagement, relationships, meaning, and accomplishment), health, and general happiness and the overall score are positive, while the correlation between negative emotions, loneliness, and the overall score was significant and negative. This indicates that there is considerable internal consistency between the factors of the PERMA Profile and the overall well-being score.

### 3.2. Confirmatory Factor Analysis (CFA)

Confirmatory factor analysis was applied to evaluate the structural validity of the scale. It answered the following critical question: Do the five PERMA domains empirically function as indicators of a single overarching well-being factor in the Saudi population? This section includes model fit indices to determine whether the observed data align with the theoretical model; factor loadings that show the strength of each domain’s contribution to the latent construct; and R^2^ values that quantify how much variance in each domain is explained by overall well-being. Placing CFA after internal consistency follows standard psychometric validation procedures and mirrors best practices in psychological measurement, ensuring that item-level coherence supports a valid latent structure.

**Figure 2:**
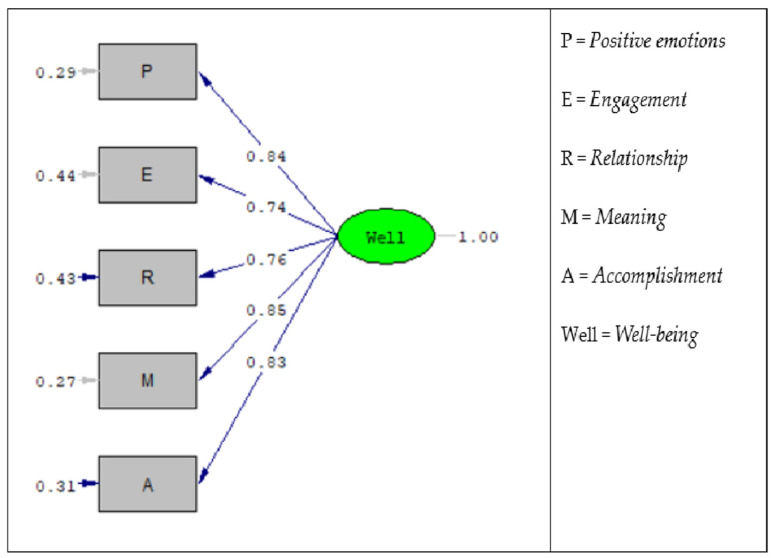
A confirmatory factor analysis model for the PERMA profiler in the Saudi context.

The factor validity or latent construction validity of the well-being scale was verified using confirmatory factor analysis for a sample of 2927 participants, in which it was assumed that the five factors within the scale were organized around one latent factor, which was well-being (see Figure 2). The numbers associated with each arrow in the diagram represent Loadings or validity coefficients after performing model calculations using the LISREL package (version 8.80). The one-factor confirmatory factor analysis (CFA) model (Figure 1) treated the five PERMA domains as observed indicators of a single latent well-being factor. The standardized factor loadings were high and statistically significant; these included meaning (λ = 0.85), positive emotions (λ = 0.84), accomplishment (λ = 0.83), relationships (λ = 0.76), and engagement (λ = 0.74). These loadings indicate that the latent well-being factor is a strong predictor of variance in all five indicators. The arrows entering the scale dimensions from the left side represent the error variance, which is calculated using the following equation:

Error Variance = 1 − (loading Square)

The single latent factor model of well-being achieved a good statistical fit, as supported by the indices (CFI = 0.999, TLI = 0.999, GFI = 0.998, NFI = 0.999, RFI = 0.998, IFI = 0.999, RMSEA = 0.022, SRMR = 0.006). NFI, GFI, TLI, CFI, RFI, and IFI indices with values from 0.90 to 1 indicate a good fit, while values from 0.80 to less than 0.90 indicate an acceptable fit; SRMR and RMSEA indices with values from 0 to 0.05 indicate a good fit, and values of more than 0.05 to 0.08 indicate an acceptable fit [37,38]. Here, (χ^2^ = 11.943, df = 5 and *p* = 0.036) indicate that the model is not a good fit, but Kline [39] pointed out that the Chi-Square index is sensitive to sample size, and that when the sample is very large (as it is here, where it is equal to 2927), the Chi-Square index can be unreliable and may indicate that the model is not a good fit, even if the inverse is true. As such, it is better to use other good fit indices such as RMSEA, CFI, TLI, etc.

Table 4 indicates that all the validity coefficients or loadings of well-being factors with a latent single factor of the well-being scale are statistically significant, which supports the validity of all factors of the PERMA profiler. The reliability coefficients of the five factors of the PERMA profiler are fairly high, ranging from 0.55 to 0.72. These are acceptable coefficients that showcase the reliability of all factors of the PERMA profiler. The confirmatory factor analysis provided strong evidence of the latent structural validity of this instrument, showing that the factors of well-being form a latent single factor, which the five factors or dimensions of well-being are organized around, and that these factors have an acceptable degree of reliability.

### 3.3. Reliability

The final theme addresses reliability, which examines the stability and consistency of the scale results. Whereas internal consistency focuses on item coherence and CFA tests structural validity, this section provides evidence that the questionnaire produces reliable scores across items (Pearson’s Correlation). It demonstrates Split-Half stability (Spearman–Brown), indicating that the instrument’s performance is stable even when divided into two parallel forms. Item–total correlations or alternative reliability tests (McDonald’s ω) were conducted to support the overall instrument reliability. Including reliability after CFA ensures that only a structurally validated model is then assessed for stability.

The reliability of the PERMA well-being scale for the total sample was calculated using the McDonald’s ω and Cronbach’s alpha method for the sub-factors and the total degree of the scale. The reliability was also calculated by the Spearman–Brown Split-Half method. Table 5 shows the reliability values obtained using the Cronbach’s alpha method and the Split-Half method.

It is clear from the previous table that the reliability values of Cronbach’s alpha for the factors of the PERMA Profile (positive emotions–engagement–relationships–meaning–accomplishment–negative feelings–health) reached (0.829, 0.543, 0.665, 0.836, 0.652, 0.700, and 0.787), respectively. The reliability coefficients of the same factors using the McDonald’s Omega method are higher than their counterparts using Cronbach’s Alpha, which were (0.830, 0.573, 0.679, 0.837, 0.685, 0.706, and 0.799), respectively.

The value of Spearman–Brown’s Split-Half method reached (0.840, 0.624, 0.720, 0.848, 0.731, 0.755, and 0.839), respectively. The PERMA-Profiler Cronbach’s Alpha and McDonald’s Omega coefficients demonstrated a substantial degree of internal consistency (α = 0.868, ω = 0.880). Furthermore, the Split-Half coefficient demonstrated noteworthy reliability (0.870).

The previous procedures confirmed that the PERMA profiler is reliable and valid, and can be used to measure the well-being of Saudi society. A high score on the scale indicates a high level of well-being, while a low score is indicative of a decrease.

## 4. Discussion

From the perspective of community mental health, preventive measures are always considered to be of prime importance. Therefore, the validation of the Arabic version of the PERMA profiler among the Saudi adult population could serve the purpose of measuring happiness, well-being, and healthy responses toward adjustment, as well as offering preventative effects. Overall, the PERMA-Profiler consists of twenty-three components. The Arabic translated version of the instrument exhibited favorable psychometric properties as well, maintaining the factorial structure reported in the original version [15]. The five factors comprising the instrument are correlated, which is consistent with Seligman’s PERMA model [10]. Pearson’s Correlation and Spearman–Brown values exhibit promising indications of reliability, albeit concisely. As demonstrated by these indices, this apparatus is short, straightforward, and dependable. The overall score itself plays a role in providing a sufficient assessment of the construct of global well-being. The ramifications of these observations and possible explanations will be discussed in the next section.

The outcomes, as mentioned above, collectively support the hypothesis that the Arabic language translated PERMA well-being scale is a reliable and valid instrument for assessing the Saudi sample’s well-being. This supports the cross-cultural validity of the PERMA model in non-Western settings. The Arabic version of the instrument also exhibits favorable psychometric characteristics. Its factorial structure remains unchanged from the original [15]; its five factors are correlated, consistent with Seligman’s PERMA model, which states that these five domains can be defined and assessed as distinct yet interrelated constructs [10]. Consequently, the initial iteration of the PERMA-Profiler included the following five subscales: (1) accomplishment, (2) positive emotions, (3) engagement, (4) relationships, and (5) meaning. Prior research has demonstrated the applicability of these dimensions when evaluating the overall well-being of individuals. Similarly, Negovan [40] reported that psychological engagement, the meaning of life, and social well-being emerged as outcomes among Romanian students’ psychosocial well-being.

Confirmatory factor analysis for the Saudi adult population provided further support for the proposed five-factor model in assessing general well-being. Similarly, Ryff and Keyes [41] examined the theoretical model of psychological well-being in the United States, which comprises six distinct dimensions of well-being (engagement, environmental mastery, personal growth, positive relationships with others, meaning of life, and self-acceptance). Linton et al. [42] argued for six important thematic domains, including meaning of life, social well-being, physical well-being, spiritual well-being, participation, and personal circumstances as central characteristics of well-being scales in an evaluation of 99 self-report measures for assessing adult well-being.

The psychometric evaluation results of the PERMA profiler for the Saudi adult population were very consistent with the results of the Brazilian adult population in terms of the internal consistency of all the factors [26]. At the same time, the results of an acceptable-to-good range of scores were found for positive emotions, meaning, health, and total grade among the Saudi adult population and the Spanish sample of students and employees [25]. The results obtained for the German adult population were also closer to our findings [31].

As far as Asian countries are concerned, the results for positive emotions and meaning were found to be acceptable to good for Chinese students, similar to the findings of our study [29]. Overall, similar internal consistency was revealed between our findings and a study conducted in the Urdu language [30]. Likewise, the findings of the Turkish study were found to be relevant for all five factors [33].

Additional iterations of the PERMA profiler yielded consistent outcomes, validating the instrument’s five-factor structure. The model fit of the German version [31] was deemed acceptable; the model fit of the Greek version [28] was comparable; and the model fit of the Italian version was reasonable [14]. The Arabic version within the Palestinian population [1] demonstrated good index values, and the Portuguese version seemed to be a good model of fit values [27,43].

As far as the reliability of the PERMA profiler Arabic translated well-being scale is concerned, it yielded acceptable reliability (r = 0.85), which was fully comparable to the original form [15], and it also provided validation in other cultural contexts. This outcome substantiates the consistency of Arabic translation. Our findings indicate that the PERMA subscales, including the supplementary domains of health and negative emotions, exhibited high reliability coefficients. Conversely, the Cronbach’s alphas for the following subscales—positive emotions (3 items, α = 0.829), relationships (3 items, α = 0.665), meaning (3 items, α = 0.836), accomplishment (3 items, α = 0.652), and overall well-being (15 items, α = 0.868)—exhibited moderate-to-strong internal consistency.

In contrast, the Arabic version of the instrument had the most significant difficulty with the constructs of engagement. The internal consistency of the engagement subscale was inadequate (three items, α = 0.543). The previous results for validation studies of PERMA profiler identified less reliability for the engagement subscale from Brazil, Spain, Turkey, and Portuguese, ranging from 0.46 to 0.69. This indicates some serious issues with this subscale and warrants detailed item analysis, which could provide additional insight into which specific items within the subscales may have contributed to lower reliability scores. Identified problematic items could inform future revisions of the scale [14,15,25,26,27,33,43,44]. This can be explained by the contextual heterogeneity of the domain and the concise architecture of the PERMA profiler (i.e., three items per domain). Internal consistency indices, including Cronbach’s alpha, increase as the number of items used increases [45]. Due to its complexity, Butler and Kern [15] emphasized the difficulties of assessing engagement as three-dimensional (cognitive, behavioral, and emotional); it is challenging to assess using a brief scale consisting of only three items. The literature indicates that engagement is a state of emotional involvement that is associated with numerous favorable consequences; nevertheless, the academic community has yet to provide a specific definition for this notion [46]. The concept and understanding of engagement might be influenced by cultural values. One of the reasons for the underscored engagement factor could be linked to cultural aspects embedded with the burden of financial competition, rapidly changing technologies, and high-standard futuristic goals. The majority of participants were young (aged 18–24), which might be linked to an immature attitude toward the engagement factor. Young people generally require more institutional support and rigorous feedback to keep them engaged [47].

## 5. Theoretical and Practical Implications

The implications of the current study’s results extend beyond the advancement of well-being research in Saudi Arabia in accordance with a multidimensional framework for therapeutic practice in psychotherapy and counseling. Counselors and psychotherapists may employ the PERMA profiler not only for screening purposes but also to assess the overall efficacy of therapy session outcomes, case studies, and evidence-based research. Additionally, PERMA profiler can be used to create interventions in a variety of settings, including universities, colleges, companies, and prisons. It could serve as a preventive measure for community health promotion campaigns. Moreover, it can contribute to the global discourse on the multifaceted character of well-being and cross-cultural research on this concept.

## 6. Strengths, Limitations, and Future Directions

This research endeavor enabled the validation of the Arabic iteration of a widely employed instrument in the field of well-being research. A multitude of interventions have been developed in clinical, educational, and occupational settings with the PERMA paradigm in mind. As a result, validating and adapting the PERMA-Profiler for an Arabic context enables the development of a multidimensional well-being instrument that may serve as a valuable indicator of changes in positive functioning and facilitate the evaluation of well-being programs in the Saudi population. This study validates the acceptance of the five-factor model across various demographic groups, ages, and genders. Moving forward, it will be imperative to persist with the validation of multi-component programs that aim to advance several facets of societal well-being. Among the study’s strengths is the sizeable sample size, which includes individuals who completed the PERMA profiler to measure their well-being.

This study had certain restrictions. One primary constraint pertains to the participant sample, which exhibited a predilection towards highly educated women. Thus, gender proportional representation can be addressed in future studies. Despite the broad age range and confirmation of the model in a sample comprising individuals of varying academic and socioeconomic standings, and differing age profiles, it remains conceivable that this sample fails to represent individuals with a comparatively lower socioeconomic status or educational attainment. To increase the scope of this scale’s application to further contexts, validation studies involving samples of various ages, genders, socioeconomic backgrounds, professions, and populations, including senior citizens, will be required in the future. This can be achieved by using cross-sectional studies. Secondly, causality and direction were not established in the interactions with other well-being factors (e.g., performance, adjustment, satisfaction, adaptability, and stress management). Consequently, longitudinal research would be advantageous. The third issue was that the engagement component lacked reliability. Therefore, a qualitative method of measurement would help to elaborate on the engagement factor among the Saudi population. Further, interviews and an examination of cultural differences regarding engagement should be conducted to assess the level of comprehension of the items comprising this dimension. This could help to reduce self-report biases.

## 7. Conclusions

In conclusion, we concur that the Arabic-translated PERMA profiler is a valid and reliable tool for assessing the well-being of the Saudi adult population. However, no single perfect PERMA profile exists, as different profiles may vary in their ability to adapt to the specific needs and circumstances of different individuals. Moreover, our findings provide a platform to utilize the profiler for counseling and mental health services. This study also supports the exploration and development of specific psychotherapeutic strategies and community-based preventive interventions to enhance the compromised domain of engagement. The connections between various profiles and other aspects of health or adaptation to particular circumstances should also be investigated.

## Figures and Tables

**Table 1:** Data from studies which have adapted and translated PERMA.

Language of Translation	Sample Size	Statistical Analysis	Results	Cronbach’s Alpha
Spanish study [25]	23,723 university students 2783 employees	Factor, Reliability, Validity Analysis	The results showed acceptable reliability and good convergent and discriminant validity between well-being variables.	The values of Cronbach’s alpha for sample 1 and sample 2: Positive emotions: Sample 1: 0.88; Sample 2: 0.90 Engagement: Sample 1: 0.69; Sample 2: 0.65 Relationship: Sample 1: 0.77; Sample 2: 0.75 Meaning: Sample 1: 0.87; Sample 2: 0.88 Accomplishment: Sample 1: 0.80; Sample 2: 0.76 Total PERMA-15: Sample 1: 0.94; Sample 2: 0.92 Negative emotions: Sample 1: 0.77; Sample 2: 0.76 Health: Sample 1: 0.86; Sample 2: 0.87 Total PERMA23: Sample 1: 0.90; Sample 2: 0.91.
Brazilian study [26]	1327 adults	Confirmatory factor analysis and correlations between subjective well-being and gratitude, optimism, self-esteem, and happiness	Positive correlations between PERMA and subjective well-being, psychological well-being, gratitude, optimism, self-esteem, and happiness were found. The adapted version of the scale showed satisfactory validity evidence based on content, internal structure, and relationships with other variables, making the scale reliable for use in Brazil.	Cronbach’s alpha coefficients showed internal consistency for all factors, ranging from 0.90 to 0.76, except for the Engagement factor, in which the alpha was 0.59. The Guttman and McDonald reliability indexes were calculated for all PERMA dimensions and showed the following results: Positive emotion—λ6 = 0.86; McDonald Ω = 0.90, Engagement—λ6 = 0.52 and Ω = 0.62, Relationship—λ6 = 0.71 and Ω = 0.78, Meaning—λ6 = 0.80 and Ω = 0.86, Accomplishment—λ6 = 0.76 and Ω = 0.81. The general PERMA factor (15 PERMA items plus the general happiness item) presented λ6 = 0.94 and Ω = 0.94.
Portuguese study [27]	434 senior tourists	Reliability Confirmatory analysis	The results revealed that the experiences of senior tourists when visiting the island of São Miguel contributed significantly to their well-being, and the modified model presented superior adjustment quality.	The values of Cronbach’s alpha were considered unacceptable for the dimensions Engagement = 0.46; Relationship = 0.59, low for the dimensions Accomplishment = 0.66 and Positive Emotions = 0.77. Moderate for the Meaning dimension = 0.82.
Greek study [28]	2539 participants	Factor structure, measurement invariance, reliability, and convergent and discriminant validity	The results demonstrated acceptable internal consistency and test–retest reliability for the overall well-being items and for almost all well-being components. The Greek version of PERMA profiler demonstrated good convergent validity with several well-being indices and discriminant validity with psychological symptoms and the experiencing of negative emotions.	Cronbach’s alpha ranged from 0.60 to 0.95.
Chinese study [29]	309 university students	Exploratory factor analysis Confirmatory factor analysis	The findings highlight the significance of validating and confirming the PERMA structure and provide new insights into its application across different cultural contexts.	Cronbach’s alphas of each subconstruct are as follows: Positive emotions, α = 0.853; engagement, α = 0.871; relationship, α = 0.953; meaning, α = 0.924 and accomplishment, α = 0.902. All the CR values for the subconstructs of PERMA exceeded the desirable standard of 0.60, which indicated high internal consistency. The AVE for the five latent variables exceeded the common cut-off value of 0.50, demonstrating that this research had acceptable discriminant validity.
Urdu Study [30]	600 students (18–26 years)	Confirmatory factor analysis Reliability	The findings showed that the Urdu version of the PERMA profiler has strong psychometric properties, is linguistically and culturally acceptable, and paves the way for music psychology to make research available so that the construct can be measured indigenously.	Cronbach’s alphas of English and Urdu versions for PERMA profiler were 0.78 and 0.81, respectively.
German study [31]	854 participants (aged from 18 to 65)	Confirmatory factor analysis of the reliability (internal consistency) Cronbach’s alpha coefficient (α) and Guttman’s lambda 6 (λ6)	The results provide evidence of acceptable reliability, very good construct validity (factorial and convergent), and first indications for measurement invariance, for both gender and nationality.	Cronbach’s alpha values ranged from 0.70 to 0.95.
Korean study [32]	316 Korean workers	High convergence, divergence, structural validity, and reliability	The results showed that the Workplace PERMA-Profiler has good convergent and divergent validity.	Cronbach’s alpha values for the Korean Workplace PERMA-Profiler ranged from 0.70 to 0.95.
Turkish, 3 studies [33]	84 university students 250 university students 272 university students	Reliability Confirmatory analysis	There is sufficient evidence that the 23-item Turkish form of the well-being scale can measure the patience of individuals according to seven basic dimensions.	The Cronbach’s alpha coefficients of the Turkish form of the scale vary between 0.62 and 0.82. The Cronbach’s alpha internal consistency coefficients for each subscale are as follows: Positive Emotion α = 0.77, Engagement α = 0.62, Relationship α = 0.70, Meaning α = 0.82, Accomplishment α = 0.70, Negative Emotion α = 0.65, Health α = 0.83. The internal consistency coefficient for the entire scale is α = 0.82.
Japanese study [34]	310 (baseline) and 86 (follow-up) workers	Reliability Convergent validity	Well-being and the five PERMA domains have moderate-to-strong correlations with job satisfaction, psychological distress (inversely), and work-related factors.	Cronbach’s alpha coefficients ranged from 0.75 to 0.96.

**Table 2:** Characteristics of the sample by demographic variables (n = 2927).

Variable	Category	N	%
Sex	Males	880	30.1
	Females	2047	69.9
Nationality	Saudi	2734	93.4
	Non-Saudi	193	6.6
Type of Accommodation	Rent	1083	37
	Owned	1844	63
Special needs	Yes	29	1
	No	2898	99
Age	18–24	1305	44.6
	25–34	683	23.3
	35–49	725	24.8
	50–65	191	6.5
	More than 65	23	0.8
Education	Higher than University	185	6.3
	University	1969	67.3
	Secondary	658	22.5
	Intermediate	72	2.5
	Primary	23	0.8
	Illiterate	20	0.7
Income level (Average monthly)	Less than SAR 15,000	1862	63.6
	Between SAR 15,000 AND SAR 25,000	612	20.9
	More than SAR 30,000	136	4.6
	Other	317	10.8
Employment status	Employed	1107	37.8
	Unemployed	562	19.2
	Student	1142	39
	Retired	116	4
Family size	1–2 members	291	9.9
	3–4 members	577	19.7
	More than (5)	2059	70.3
Marital status	Single	1630	55.7
	Married	1186	40.5
	Divorced	85	2.9
	Widowed	26	0.9

**Table 3:** The internal consistency of the items and subscales of the PERMA profile among the study sample (n = 2927).

Subscales	Items	Correlation with Subscales	Correlation with the Overall Well-Being Score	
Positive emotions	P1	0.873 **	0.691 **	0.825 **
	P2	0.866 **	0.737 **	
	P3	0.853 **	0.715 **	
Engagement	E1	0.766 **	0.637 **	0.785 **
	E2	0.752 **	0.723 **	
	E3	0.653 **	0.350 **	
Relationships	R1	0.745 **	0.526 **	0.769 **
	R2	0.774 **	0.621 **	
	R3	0.816 **	0.655 **	
Meaning	M1	0.874 **	0.737 **	0.837 **
	M2	0.873 **	0.731 **	
	M3	0.857 **	0.712 **	
Accomplishment	A1	0.821 **	0.675 **	0.797 **
	A2	0.813 **	0.623 **	
	A3	0.662 **	0.534 **	
Negative emotions	N1	0.786 **	0.099 **	−0.069 **
	N2	0.766 **	0.370 **	
	N3	0.101 **	0.097 **	
Health	H1	0.778 **	0.579 **	0.686 **
	H2	0.863 **	0.571 **	
	H3	0.873 **	0.582 **	
Feelings of loneliness	Lon	-	0.582 **	0.582 **
Happiness	Hap	-	0.711 **	0.711 **

** Significant at (0.01).

**Table 4:** The loading of well-being factors with a latent single factor of the well-being instrument; (T) values and standard error of loading estimation and coefficient and stability.

No.	Factors of Well-Being	Loading	The Standard Error of Loading Estimation	T-Value	Significance Level	Coefficient of Reliability R^2^
1	Positive emotions	0.84	0.015	55.11	0.01	0.71
2	Engagement	0.74	0.016	46.27	0.01	0.55
3	Relationship	0.76	0.017	44.96	0.01	0.58
4	Meaning	0.85	0.015	55.73	0.01	0.72
5	Accomplishment	0.83	0.016	51.45	0.01	0.69

**Table 5:** Reliability coefficients obtained for the PERMA scale using Cronbach’s alpha and Spearman–Brown’s Split-Half method.

Factors	Number of Items	Cronbach’s Alpha	McDonald’s ω	Spearman–Brown’s Split-Half Method
Positive emotions	3	0.829	0.830	0.840
Engagement	3	0.543	0.573	0.624
Relationships	3	0.665	0.679	0.720
Meaning	3	0.836	0.837	0.848
Achievement	3	0.652	0.685	0.731
Negative emotions	3	0.700	0.706	0.755
Health	3	0.787	0.799	0.839
Total Grade	23	0.868	0.880	0.870

Note: McDonald’s ω HA was used for the subscales, while McDonald’s ω ML was used for the scale as a whole.

## Data Availability

The datasets analyzed within this study can be found in the OSF REPOSITORY [https://osf.io/8srkc/ (accessed on 10 March 2024)] upon request.

## Abbreviations

The following abbreviations are used in this manuscript:

PERMA model	Positive Emotion, Engagement, Relationships, Meaning, and Accomplishment model

## Appendix A

Back Matter:

A. Notes on Sampling Approach
  - Stratified sampling ensured geographic proportionality across the 13 regions.
  - Additionally, saturation was employed within each stratum during the data-saturation phase.
B. Notes on Data Exclusion
  - Incomplete surveys were excluded using a single exclusion rule: fewer than 80% of items completed.
C. Notes on Statistical Power
  - Maintaining a final sample >2500 ensured a margin of error ≤2% (98% confidence).
